# The *p22phox* Polymorphism C242T Is Not Associated with Peripheral Malondialdehyde and Superoxide Dismutase Levels in Healthy Korean Adults

**Published:** 2019-05

**Authors:** Su-Youn CHO, Wi-Young SO, Hee-Tae ROH

**Affiliations:** 1. Department of Physical Education, College of Science in Education, Yonsei University, Seoul, Korea; 2. Sports and Health Care Major, College of Humanities and Social Sciences, Korea National University of Transportation, Chungju-si, Korea; 3. Department of Physical Education, College of Arts and Physical Education, Dong-A University, Busan, Korea

## Dear Editor-in-Chief

NADPH oxidase consists of six subunits: p47phox, p67phox, p40phox, and Rac in the cytoplasm combined with gp91phox and p22phox in the cell membrane (1). Among these six subunits, p22phox is found in the NADPH oxidase of neutrophils and vascular smooth muscle cells. The p22phox subunit has been shown to play a role in reactive oxygen species (ROS) generation in blood and blood vessel cells as a component of flavocytochrome b558 (1). Decreasing p22phox expression entails a markedly reduced level of ROS, thus supporting the above observation (2). Ten polymorphic genes encode p22phox (3). The locus of *p22phox* is found on chromosome 16q24, and the specific C242T polymorphic genes involved in the substitution of histidine by tyrosine have been reported as determinants of superoxide anion (O_2_^−^) generation (4). In other words, among the three alleles of C242T (CC, TC, and TT), the saphenous vein of an individual with the T allele (TC+TT genotype) shows lower O_2_^−^ than that of an individual with the CC genotype, as reported in a study on the C242T polymorphism in the NADPH oxidase *p22phox* gene (4). To further investigate this, a number of previous studies have assessed the incidence of type II diabetes and cardiovascular diseases based on the C242T polymorphism, and a correlation has been confirmed (5). However, few studies have reported the levels of oxidative stress associated with C242T polymorphism in the *p22phox* gene in healthy subjects, and a recent meta-analysis implied that the variation could be due to race (6).

Thus, the aim of the present study was to verify differences in oxidative stress between the CC genotype and the T allele by investigating the C242T polymorphism in the NADPH oxidase *p22phox* gene in healthy Korean individuals and analyzing the two plasma indices, malondialdehyde (MDA) and superoxide dismutase (SOD), which determine the oxidative/anti-oxidative status in the body.

To investigate the C242T polymorphism in the NADPH oxidase *p22phox* gene, 200 healthy Korean individuals (100 men and 100 women) were evaluated, and 20 individuals with the CC genotype (10 men and 10 women) and 20 individuals with the T allele (10 men and 10 women), who did not smoke or drink and had normal body composition, were selected.

The study conformed to the standards set by the latest revision of the Declaration of Helsinki. All participants read and signed a written informed consent statement consistent with the guidelines set by the Department of Physical Education at Yonsei University.

The body composition was estimated using bioelectrical impedance analysis (M310; Biodynamic, USA). The C242T polymorphism in the NADPH oxidase *p22phox* gene and plasma MDA and SOD levels were analyzed using the methods described below.

To determine the genotype of the C242T polymorphism in the NADPH oxidase *p22phox* gene, genomic DNA was extracted from 3 mL whole blood using a DNA isolation kit (Gentra Genomic DNA Purification Kit, USA) following the protocol provided by the manufacturer. The analyses of plasma MDA and SOD were carried out using a BIOXYTECH LPO-586 kit (Oxis, USA) and a tetrazolium-based kit (IBL, Germany), respectively.

Table 1 shows the anthropometric and biochemical characteristics of the participants according to the C242T polymorphism in the NADPH oxidase *p22phox* gene. Statistical analysis showed that there were no significant differences between groups in age, height, weight, body mass index, body fat, and lean body mass (*P*>0.05). In particular, no statistically significant differences were found in plasma MDA and SOD levels, the two indices of oxidative stress (*P*>0.05). The C242T polymorphism in the NADPH oxidase *p22phox* gene in healthy Korean adults did not affect the level of oxidative stress when the individuals are at rest.

**Table 1:** Anthropometric and biochemical characteristics of the participants

***Variables/Group***	***CC genotype (n=20)***	***T allele (n=20)***	***P-value***
Age (yr)	23.65± 2.50	22.30± 2.03	0.068
Height (cm)	169.80± 8.04	171.10± 8.57	0.622
Weight (kg)	63.35± 11.07	65.72± 9.05	0.463
Body mass index (kg/m^2^)	21.82± 2.31	22.38± 1.88	0.405
Body fat (%)	20.43± 7.12	21.98± 6.97	0.488
Lean body mass (kg)	45.26± 18.09	43.39± 17.77	0.743
Malondialdehyde (nmol/mL)	5.36± 0.88	5.29± 1.08	0.824
Superoxide dismutase (U/mL)	3.22± 0.42	3.21± 0.43	0.920

Data are presented as mean ± standard deviation.

Tested by independent t-tests

Further studies should investigate the correlations between the C242T polymorphism in the NADPH oxidase *p22phox* gene and the level of oxidative stress by inducing a transient increase in oxidative stress after high-intensity acute exercise.
